# Short-Term Plasticity Regulates Both Divisive Normalization and Adaptive Responses in *Drosophila* Olfactory System

**DOI:** 10.3389/fncom.2021.730431

**Published:** 2021-10-22

**Authors:** Yuxuan Liu, Qianyi Li, Chao Tang, Shanshan Qin, Yuhai Tu

**Affiliations:** ^1^School of Physics, Peking University, Beijing, China; ^2^Integrated Science Program, Yuanpei College, Peking University, Beijing, China; ^3^Biophysics Graduate Program, Harvard University, Cambridge, MA, United States; ^4^Center for Quantitative Biology, Peking University, Beijing, China; ^5^Peking-Tsinghua Center for Life Sciences, Peking University, Beijing, China; ^6^John A. Paulson School of Engineering and Applied Sciences, Harvard University, Cambridge, MA, United States; ^7^Physical Sciences Department, IBM T. J. Watson Research Center, Yorktown Heights, NY, United States

**Keywords:** short-term plasticity, presynaptic inhibition, circuit model, olfactory system, *Drosophila*, divisive normalization

## Abstract

In *Drosophila*, olfactory information received by olfactory receptor neurons (ORNs) is first processed by an incoherent feed forward neural circuit in the antennal lobe (AL) that consists of ORNs (input), inhibitory local neurons (LNs), and projection neurons (PNs). This “early” olfactory information processing has two important characteristics. First, response of a PN to its cognate ORN is normalized by the overall activity of other ORNs, a phenomenon termed “divisive normalization.” Second, PNs respond strongly to the onset of ORN activities, but they adapt to prolonged or continuously varying inputs. Despite the importance of these characteristics for learning and memory, their underlying mechanisms are not fully understood. Here, we develop a circuit model for describing the ORN-LN-PN dynamics by including key neuron-neuron interactions such as short-term plasticity (STP) and presynaptic inhibition (PI). By fitting our model to experimental data quantitatively, we show that a strong STP balanced between short-term facilitation (STF) and short-term depression (STD) is responsible for the observed nonlinear divisive normalization in *Drosophila*. Our circuit model suggests that either STP or PI alone can lead to adaptive response. However, by comparing our model results with experimental data, we find that both STP and PI work together to achieve a strong and robust adaptive response. Our model not only helps reveal the mechanisms underlying two main characteristics of the early olfactory process, it can also be used to predict PN responses to arbitrary time-dependent signals and to infer microscopic properties of the circuit (such as the strengths of STF and STD) from the measured input-output relation. Our circuit model may be useful for understanding the role of STP in other sensory systems.

## 1. Introduction

Sensory systems have evolved different strategies to efficiently represent and process physiologically relevant stimuli in the presence of various biophysical constraints. For example, the olfactory system is confronted with the challenge that there are numerous odors each consisting of multiple volatile molecules with a wide range of concentrations. Yet the olfactory system possesses a remarkable ability to detect and discriminate odors using a relatively small repertoire of odor receptors (ORs) through a combinatorial code, i.e., each odorant is sensed by multiple receptors and each receptor can be activated by many odorants (Hallem and Carlson, 2006; Saito et al., 2009; Si et al., 2019).

The functional organization of the olfactory systems across different species is highly conserved (Su et al., 2009; Hansson and Stensmyr, 2011; Uchida et al., 2014). In both insects and vertebrates, an olfactory receptor neuron (ORN) typically expresses only one type of OR. ORNs that express the same OR converge to the same glomerulus in the olfactory bulb (vertebrates) or the antennal lobe (AL, insects). In *Drosophila*, peripheral odor information is processed in the AL before transmitted to higher brain areas by projection neurons (PNs) (Masse et al., 2009; Wilson, 2013). Each PN typically innervates one glomerulus. The transfer function between ORN and PN is a saturating nonlinear function (Bhandawat et al., 2007; Kazama and Wilson, 2008; Olsen and Wilson, 2008), i.e., a small ORN input is disproportionally amplified while a strong input saturates the response. Lateral inhibition by local interneurons (LNs) in the AL increases the level of ORN input needed to drive PNs to saturation, the strength of inhibition scales with the total forward input to the AL, a phenomenon called “divisive normalization” (Olsen et al., 2010; Carandini and Heeger, 2012), which has been widely observed across different sensory modalities and brain regions (Carandini and Heeger, 2012; Ferguson and Cardin, 2020). Divisive normalization in the AL was found beneficial for efficient odor coding (Olsen and Wilson, 2008; Luo et al., 2010).

Airborne odors are intermittent and have complex spatio-temporal profiles (Murlis et al., 1992; Vickers et al., 2001). The ability to detect and respond to temporal variation of odors is crucial for successful odor-guided navigation (David et al., 1983; Victor et al., 2019; Demir et al., 2020). This is partially achieved by the adaptive responses of PNs to time-dependent inputs from ORNs. In particular, PNs respond transiently to the onset of a step-function like ORN input and fall back to low firing rates for the prolonged input, showing highly adaptive response. For more complex time-dependent ORN inputs, the response of PNs depends on both the ORN firing rate and its rate of change (Kim et al., 2011, 2015). Indeed, one of the hallmark properties of all sensory systems is adaptation, which is crucial for detecting and tracking time-varying signals (Wark et al., 2007). For adaptive response in *Drosophila* olfactory system, although a phenomenological linear-nonlinear model was proposed to fit experimental data (Kim et al., 2015), a mechanistic understanding of how the ORN-PN-LN circuit in the AL leads to the adaptive response is still missing.

The aim of this study is to understand the mechanistic origins of both divisive normalization and adaptive response in *Drosophila* by modeling dynamics of the AL neural circuit. From previous studies (Kazama and Wilson, 2008; Martelli and Fiala, 2019), the synapses between ORNs and PNs in AL exhibit strong short-term plasticity (STP), which is a form of fast activity-dependent modulation of synaptic strength (Stevens and Wang, 1995; Abbott et al., 1997; Markram et al., 1998; Dittman et al., 2000; Wang et al., 2006). Another important factor in the AL circuit is that inhibition by LNs is due to presynaptic inhibition (PI) at the axon terminal of ORNs (Olsen and Wilson, 2008). In this paper, we develop a simple circuit model of the *Drosophila* AL that includes both STP and PI. By using analytical methods and numerical simulations, we study the effects of STP on divisive normalization and response to time-varying stimuli of PNs. From direct quantitative comparison of our model results to experimental data, we show that STP is essential for the observed highly nonlinear divisive normalization; and both STP and PI determine the adaptive response observed in experiments.

## 2. Methods

### 2.1. A Circuit Model of the Antennal Lobe With STP

**Figure 1:**
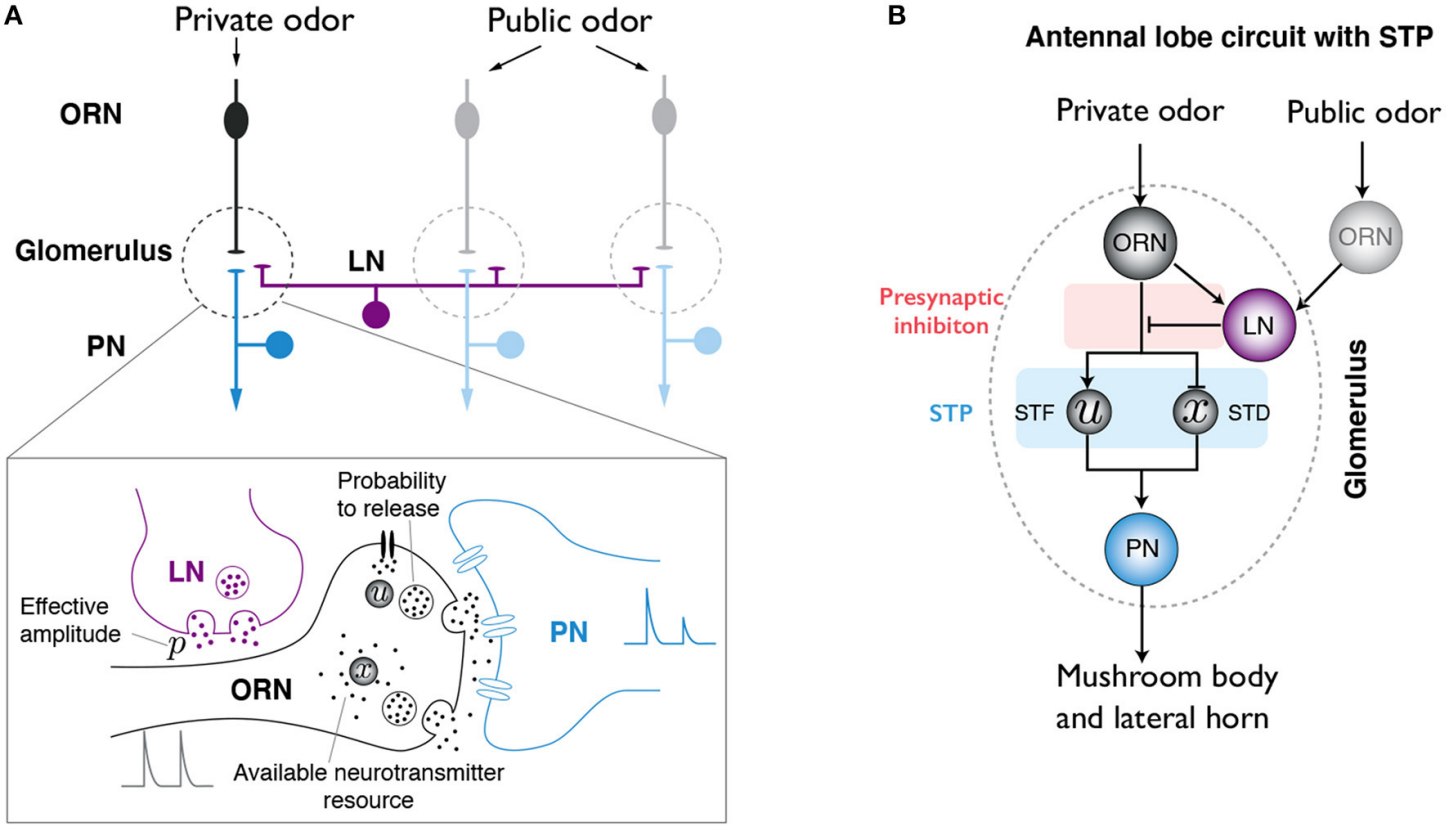
Illustration of the antennal lobe (AL) circuit model. **(A)** Simplified neural circuit in the AL of *Drosophila*. Olfactory receptor neurons (ORNs) that express the same type of olfactory receptors innervate the same glomeurlus (dashed line region) in the AL. Here, we focus on uniglomerular projection neurons (uPNs). Each of them sends dendrites into a single glomerulus and receive synaptic input from its cognate ORN. Although each glomerulus might be innervated by different PNs, only one PN is shown. Glomeuruli are laterally connected by inhibitory local neurons (LNs,magenta), which interact with ORNs and PNs. A private odor only activates a specific type of ORNs, while a public odor activates a large number of ORNs that innervate different glomeruli. Lower: close-up of the synaptic interactions between ORN, PN and LN. Both PI and STP are considered in our model. **(B)** Schematics of the AL circuit with STP effect and PI mediated by LNs.

There are around 50 types of ORNs in *Drosophila* melanogaster, each of them expresses one OR. ORNs that express the same OR converge to the same glomerulus in the AL. A given odor typically activates several types of ORNs, hence different glomeruli. Each PN innervates one glomeulus and projects to higher brain areas like mushroom body and lateral horn. Since most LNs in AL are GABAergic, we will only consider inhibitory interneurons. Although LNs have distinct morphologies, innervation patterns, and response dynamics to odors (Chou et al., 2010; Nagel et al., 2015; Nagel and Wilson, 2016), for the purpose of this study, we do not differentiate them in our model. Generally speaking, LNs innervate different glomeruli and target the boutons of ORN axons, forming presynatpic inhibition (Figure 1A). We consider the simplified neural circuit of ORN-PN-LN in the AL, as shown in Figure 1B. Since ORNs promote the firing of PNs and LNs, while LNs in turn inhibit the firing of PNs, these neurons forms an incoherent feedforward loop (IFFL), a motif that has been widely observed in biochemical networks (Shen-Orr et al., 2002; Ma et al., 2009; Tu and Rappel, 2018).

For simplicity, we used a mean-field model to describe the firing rates of the neurons (Dayan and Abbott, 2001; Trappenberg, 2009; Gerstner et al., 2014). The dynamics of PN (LN) population firing rate *R*_PN(LN)_ can be written as:

(1) $$\begin{array}{r} \frac{dR_{\text{PN}}}{dt}=-\frac{R_{\text{PN}}}{\tau_{E}}+\omega^{EE}u^{+}xpR, \end{array}$$

(2) $$\begin{array}{r} \frac{dR_{\text{LN}}}{dt}=-\frac{R_{\text{LN}}}{\tau_{E}}+\omega^{IE}\underset{j}{\sum }R_{j}, \end{array}$$

where *R* is the firing rate of the cognate ORN that responds to a particular (private) odorant. The sum $\sum_{j}$ in Equation (2) is over all ORNs connected to the LN, including non-cognate ORNs that respond only to public odorant(s). The timescale τ_*E*_ is the relaxation time of the firing rate. ω^*EE*^ and ω^*IE*^ are synaptic weights of the synapses from ORN to PN and LN, respectively, assumed to be homogeneous among different ORNs.

The effect of PI is modeled by Equation (1) with a (dimensionless) variable 0 < *p* < 1 that represents reduction of the effective ORN firing rate due to presynaptic inhibition by LN. The dynamics of *p*, simplified from previous studies (Zhang et al., 2013, 2015), is modeled as:

(3) $$\begin{array}{r} \tau_{p}\frac{dp}{dt}=-p+\frac{1}{1+\rho R_{LN}}, \end{array}$$

where ρ is a constant and τ_*p*_ is the relaxation time of *p*. In the limit τ_*p*_ ≪ τ_*E*_, we can use the quasi-steady state approximation $p\approx \frac{1}{1+\rho R_{\text{LN}}}$, which suggests that *p* decreases with *R*_LN_.

The effect of STP can be separated into short-term facilitation (STF) and short-term depression (STD), which are modeled by *u*^+^ and *x* in Equation (1), respectively. Following previous work (Tsodyks et al., 1998), we denote *u*^−^ (*u*^+^) as the neurotransmitter releasing probability just before (after) the arrival of a presynaptic spike; and *x* as the fraction of available neurotransmitters (Figure 1A). Applying the mean-field model for STP (Tsodyks et al., 1998), we have the following dynamics for *u*^−^ and *x*:

(4) $$\begin{array}{r} \frac{dx}{dt}=\frac{1-x}{\tau_{D}}-xu^{+}pR, \end{array}$$

(5) $$\begin{array}{r} \frac{du^{-}}{dt}=-\frac{u^{-}}{\tau_{F}}+U\left(1-u^{-}\right)pR, \end{array}$$

where *u*^+^ = *u*^−^ + *U*(1 − *u*^−^) with *U* as the increment in release probability after each spike. Without any presynaptic firing (*R* = 0), we have *x* = 1, *u*^−^ = 0, and *u*^+^ = *U* at steady state. With presynaptic firing, *x* decreases and *u*^+^ increases before their steady state values are recovered with time constants τ_*D*_ and τ_*F*_ respectively. The strength of STF and STD can be measured by the dimensionless recovery times $S_{F}=\frac{\tau_{F}}{\tau_{E}}$ and $S_{D}=\frac{\tau_{D}}{\tau_{E}}$ normalized by the relaxation time for firing rate τ_*E*_. The longer the STP recovery time (τ_*F*_ or τ_*D*_), the stronger the STP effect (STF or STD).

In the rest of the paper, we use the neural circuit model (Equations 1–5) to describe and explain several response properties of PNs including divisive normalization for constant (steady state) inputs and adaptive response to time-varying inputs. In both cases, we compare our model results with existing experiments and focus on understanding the effects of STP and PI on the observed behaviors.

### 2.2. Response of PNs to Triangular-Shaped Firing Rates of ORNs

In this section, we describe in detail the approximations used when we study PN's response to triangular-shaped inputs. First, we consider a simpler scenario, where ORN's firing rate increases linearly without bound, i.e., *R* = *Kt*. Then, Equations (1)–(5) become

(6) $$\begin{array}{r} \frac{dR_{LN}}{dt}=-\frac{R_{LN}}{\tau_{E}}+\omega^{IE}Kt,\\ \tau_{p}\frac{dp}{dt}=-p+\frac{1}{1+\rho R_{LN}},\\ \frac{dR_{PN}\left(t\right)}{dt}=-\frac{R_{PN}}{\tau_{E}}+\omega^{EE}u^{+}xpKt,\\ \frac{dx}{dt}=\frac{1-x}{\tau_{D}}-xu^{+}pKt,\\ \frac{du^{-}}{dt}=-\frac{u^{-}}{\tau_{F}}+U\left(1-u^{-}\right)pKt,\\ u^{+}=u^{-}+U\left(1-u^{-}\right). \end{array}$$

To analyze the adaptive behavior of PN responses, we derive an approximate solution of the above equations. These equations can be separated into two groups based on the biological mechanisms they describe: the first two equations describe presynaptic inhibition and the other equations are related to STP, which we study separately in the following.

#### 2.2.1. The Presynaptic Inhibition Equations

Solving the first two equations of Equations (6) we obtain:

(7) $$\begin{array}{r} R_{LN}\left(t\right)=\omega^{IE}K\tau_{E}\left[t-\tau_{E}\left(1-e^{-\frac{t}{\tau_{E}}}\right)\right],\\ p\left(t\right)=e^{-\frac{t}{\tau_{p}}}+e^{-\frac{t}{\tau_{p}}}\int_{0}^{t}\frac{e^{\frac{t^{\prime }}{\tau_{p}}}dt^{\prime }}{\tau_{p}\left\{1+AK\left[t^{\prime }-\tau_{E}\left(1-e^{-\frac{t^{\prime }}{\tau_{E}}}\right)\right]\right\}} \end{array}$$

with initial conditions *p*(0) = 1 and *R*_*LN*_(0) = 0. Here $A=k\rho \tau_{E}\omega^{IE}$, as defined previously in the steady state solution in the main text. Since the plateau appears at *t* ≫ τ_*E*_, the integrand of Equation (7) can be approximated as

$$\begin{array}{l} \frac{e^{\frac{t^{\prime }}{\tau_{p}}}}{\tau_{p}\left\{1+AK\left[t^{\prime }-\tau_{E}\left(1-e^{-\frac{t^{\prime }}{\tau_{E}}}\right)\right]\right\}}\approx \frac{e^{\frac{t^{\prime }}{\tau_{p}}}}{\tau_{p}\left(1-AK\tau_{E}\right)+\tau_{p}AKt^{\prime }}. \end{array}$$

The validity of this approximation is supported by direct numerical integration of Equation (7) (Supplementary Figure 6). Plugging it back into Equation (7), we obtain:

(8) $$\begin{array}{r} p\left(t\right)\approx e^{-\frac{t}{\tau_{p}}}+\frac{e^{-\left(\frac{t}{\tau_{p}}+\frac{1-AK\tau_{E}}{AK\tau_{p}}\right)}}{AK\tau_{p}}[\text{Ei}\left(\frac{t}{\tau_{p}}+\frac{1-AK\tau_{E}}{AK\tau_{p}}\right)\\ -\text{Ei}\left(\frac{1-AK\tau_{E}}{AK\tau_{p}}\right)], \end{array}$$

where $\text{Ei}\left(x\right)=\int_{-\infty }^{x}\frac{e^{x^{\prime }}}{x^{\prime }}dx^{\prime }$ denotes the exponential integral. By asymptotically expanding Ei(*x*) as $\text{Ei}\left(x\right)=\frac{e^{x}}{x}\sum_{n=0}^{N-1}\frac{n!}{x^{n}}$, we have (up to the leading order terms):

(9) $$\begin{array}{r} p\left(t\right)\approx \frac{1}{AK\left(t-\tau_{E}\right)+1}-\frac{AK\tau_{E}}{1-AK\tau_{E}}e^{-\frac{t}{\tau_{p}}}. \end{array}$$

Multiplying Equation (9) by *Kt* gives the effective input *R*_eff_ Equation (14).

#### 2.2.2. The STP Equations

The last four equations in (6) describe the STP mechanism. From Equation (9), in the limit *t* ≫ τ_*p*_, τ_*E*_, we have *p* ≈ (*AR*(*t*))^−1^, which leaves the effective input *R*_eff_ = *p*(*t*)*R*(*t*) approximately constant in time. We use this approximation to investigate the plateau behavior of PN response. Plugging it back into Equation (6), we have $u^{-}\left(t\right)=\tau_{u}UR_{\text{eff}}\left(1-e^{-\frac{t}{\tau_{u}}}\right)$ where 1/τ_*u*_ ≡ 1/τ_*F*_ + *R*_eff_*U*.

The equation for *x* now becomes:

$$\begin{array}{l} \frac{dx}{dt}+\left[\frac{1}{\tau_{x}}-\left(1-U\right)UR_{\text{eff}}^{2}\tau_{u}e^{-\frac{t}{\tau_{u}}}\right]x=\frac{1}{\tau_{D}}, \end{array}$$

where $1/\tau_{x}\equiv 1/\tau_{D}+\left(1-U\right)UR_{\text{eff}}^{2}\tau_{u}+UR_{\text{eff}}$. Introducing $P_{x}\left(t\right)=1/\tau_{x}-\left(1-U\right)UR_{\text{eff}}^{2}\tau_{u}e^{-\frac{t}{\tau_{u}}}$, we have

(10) $$\begin{array}{r} x\left(t\right)=e^{-\int_{0}^{t}P_{x}\left(t^{\prime }\right)dt^{\prime }}+e^{-\int_{0}^{t}P_{x}\left(t^{\prime }\right)dt^{\prime }}\int_{0}^{t}\frac{1}{\tau_{D}}e^{\int_{0}^{t^{\prime }}P_{x}\left(t^{\prime \prime }\right)dt^{\prime \prime }}dt^{\prime }. \end{array}$$

Since we are interested in the plateau behavior of PNs, where *t* ≫ τ_*u*_, τ_*x*_. We can approximate $\int_{0}^{t}P_{x}\left(t^{\prime }\right)dt^{\prime }$ as $\frac{t}{\tau_{x}}$. Plugging it back to Equation (10) and neglecting higher order corrections, we have *x*(*t*) ≈ τ_*x*_/τ_*D*_. Similarly,

$$\begin{array}{r} u^{+}\left(t\right)x\left(t\right)=\left(1-U\right)u^{-}\left(t\right)x\left(t\right)+Ux\left(t\right)\approx \frac{\tau_{x}}{\tau_{D}}\left[\left(1-U\right)U\tau_{u}R_{\text{eff}}+U\right]. \end{array}$$

The magnitude of the plateau response of PN can be approximated by its steady state activity for large *t*,

$$\begin{array}{l} R_{PN}\left(t\right)=\omega^{EE}\tau_{E}R_{\text{eff}}u^{+}\left(t\right)x\left(t\right)\approx \omega^{EE}\tau_{E}R_{\text{eff}}\frac{\tau_{x}}{\tau_{D}}\left[\left(1-U\right)U\tau_{u}R_{\text{eff}}+U\right], \end{array}$$

where *R*_eff_ = *p*(*t*)*Kt* ≈ 1/*A* with *p*(*t*) given by Equation (9). We see that the plateau magnitude of *R*_*PN*_ is related to *R*(*t*) only through *R*_eff_, which is the final value that *pR*(*t*) reaches. It is independent of *t* and *K*, therefore the magnitude of PN response plateau is not affected by the input changing rate *K*, but only depends on intrinsic properties of the system.

### 2.3. Determination of Model Parameters and Initial Conditions

**Figure 2:**
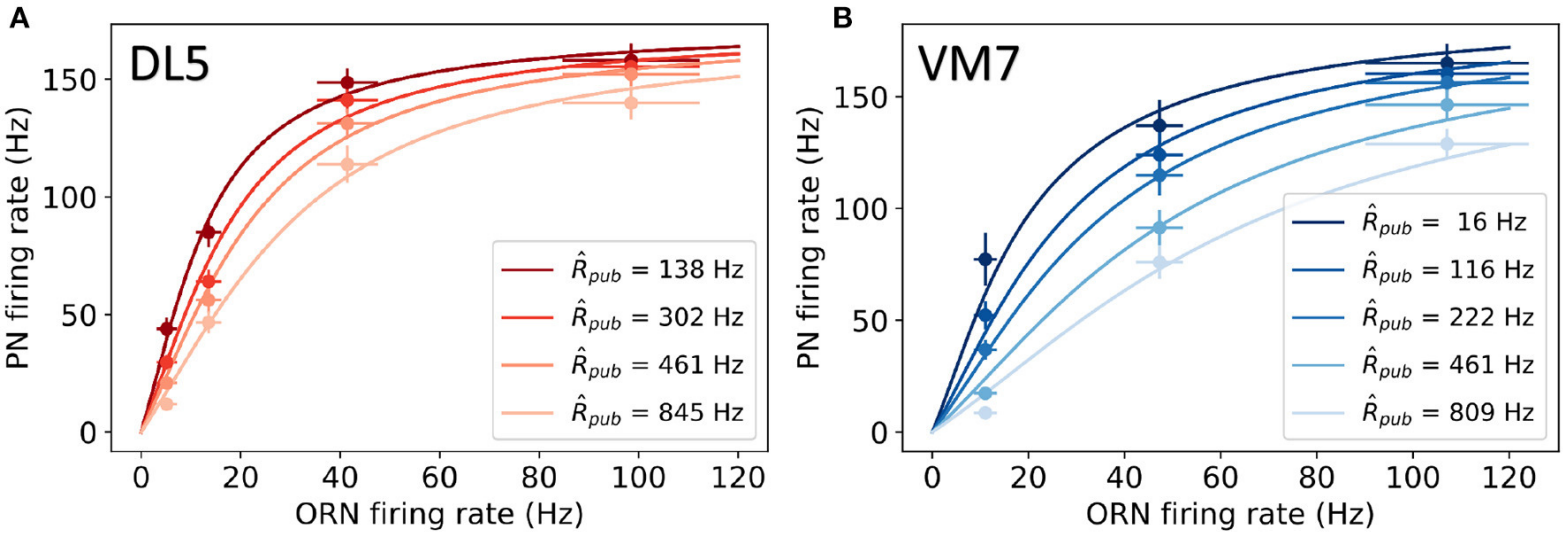
Comparison between model results (lines) and experimental data (symbols) for PNs that innervate **(A)** DL5 and **(B)** VM7 glomeruli. The experimental data are from Olsen et al. (2010). The PN firing rate shown here is averaged over the first 500 ms as done in Olsen et al. (2010). Darkness of color indicates different strengths of lateral inhibition due to different fitted public ORN firing rates ${\hat{R}}_{\text{pub}}$, which are found to be proportional to the measured local field potentials in experiments as shown in Supplementary Figure 1. Other model parameters are given in Table 1.

**Figure 3:**
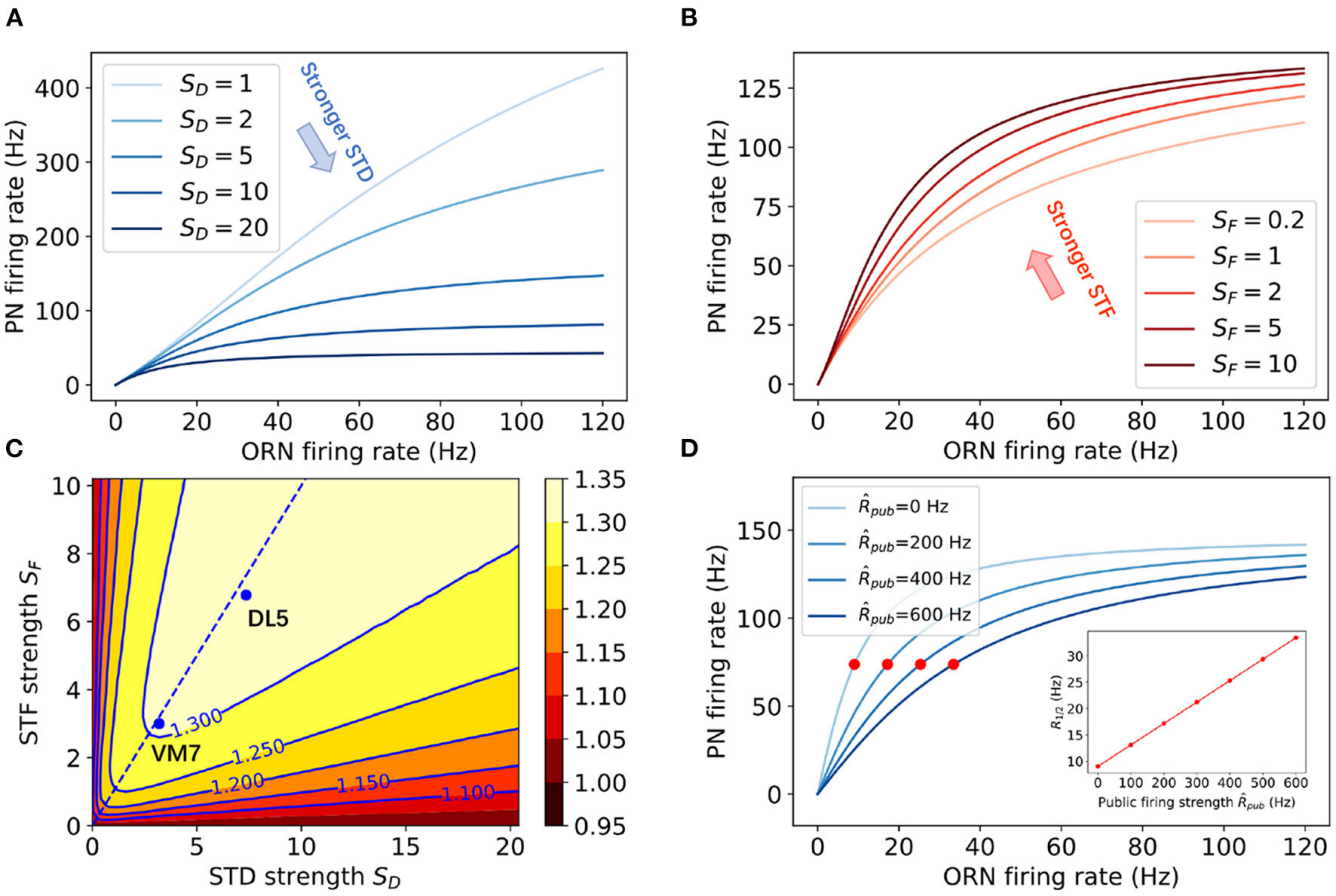
The effects of STP (STF and STD) on the nonlinear divisive normalization in PNs' responses. **(A,B)** Steady state responses of PN to different cognate ORN firing rates for **(A)** different STD strengths *S*_*D*_ and **(B)** different STF strengths *S*_*F*_. In **(A)** *S*_*F*_ = 2, in **(B)** *S*_*D*_ = 6. **(C)** The dependence of the effective Hill coefficient γ on the STF strength (*S*_*F*_) and the STD strength (*S*_*D*_). The values of *S*_*D*_ and *S*_*F*_ used in fitting the experimental data for DL5 and VM7 (see Figure 2) are also shown in the figure. The dotted line corresponds to perfectly balanced STF and STD strength: *S*_*D*_ = *S*_*F*_. **(D)** Steady state responses of PN to cognate ORN firing rates in the presence of different public firing rates ${\hat{R}}_{\text{pub}}$. Red dots mark ORN firing rates at the half maximum firing of PNs (*R*_1/2_). The inset shows that *R*_1/2_ increases linearly with ${\hat{R}}_{\text{pub}}$. Other model parameters used here are: $U=0.24,{\hat{R}}_{\text{pub}}=500\text{Hz},\omega^{\text{EE}}=180\text{nS},\omega^{IE}=10\text{nS},\tau_{E}=50\text{ms},\rho =1.8\text{ms}$.

**Figure 4:**
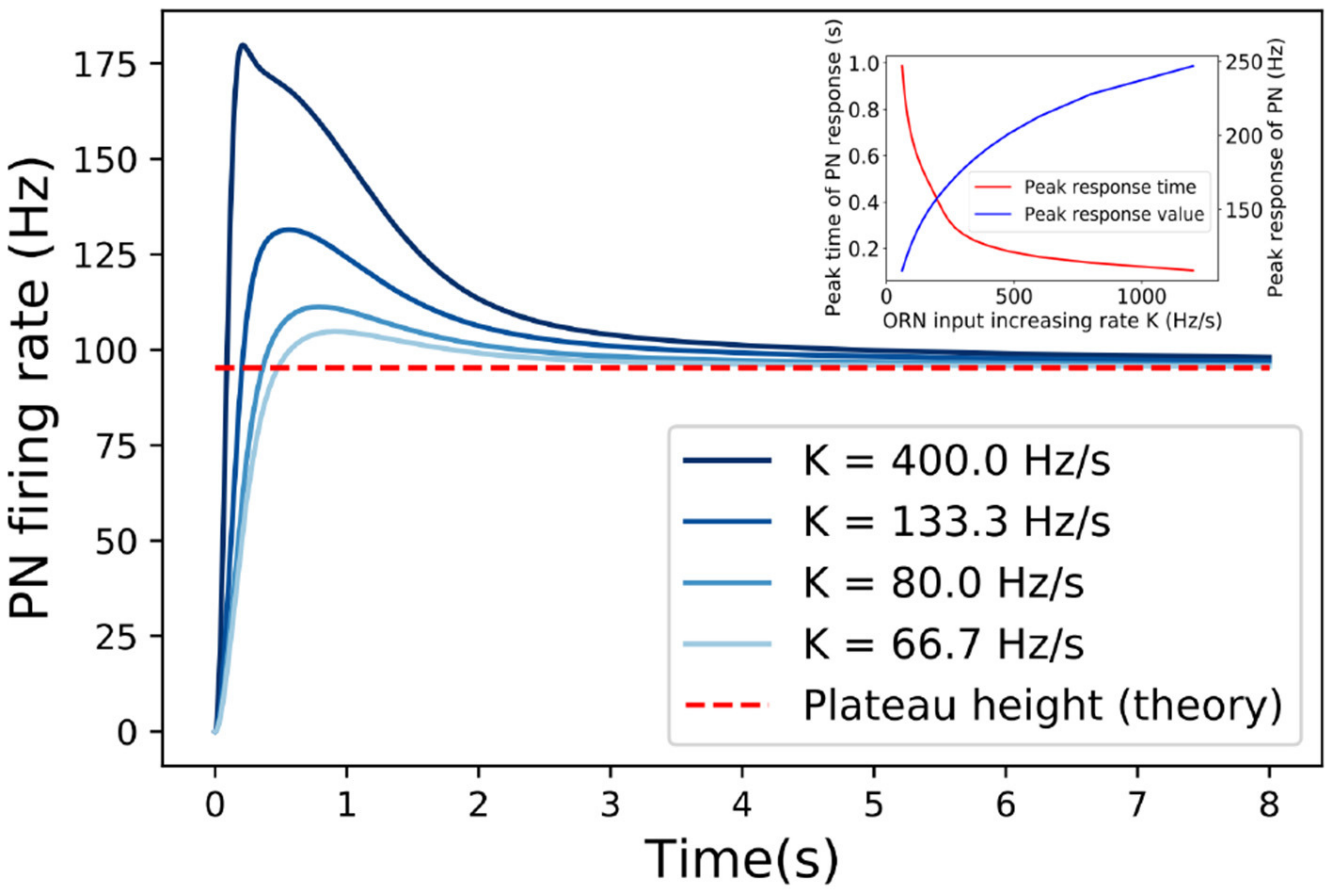
The responses of PN to linearly increasing inputs (ORN firing rate) with different increasing rate *K*. The adapted responses are independent of the increasing rate (*K*) of the input. Dashed line marks the asymptotic response when *t* → ∞ as predicted by Equation (15). The inset shows how the peak time and peak value of PN response depend on the increasing rate (*K*). Other model parameters used here are: $U=0.24,{\hat{R}}_{\text{pub}}=0,\omega^{EE}=75\text{nS},\omega^{\text{IE}}=21\text{nS},\tau_{E}=55\text{ms},\rho =8\text{ms},$ τ_*p*_ = 300ms, τ_*F*_ = 50ms, τ_*D*_ = 100ms.

**Figure 5:**
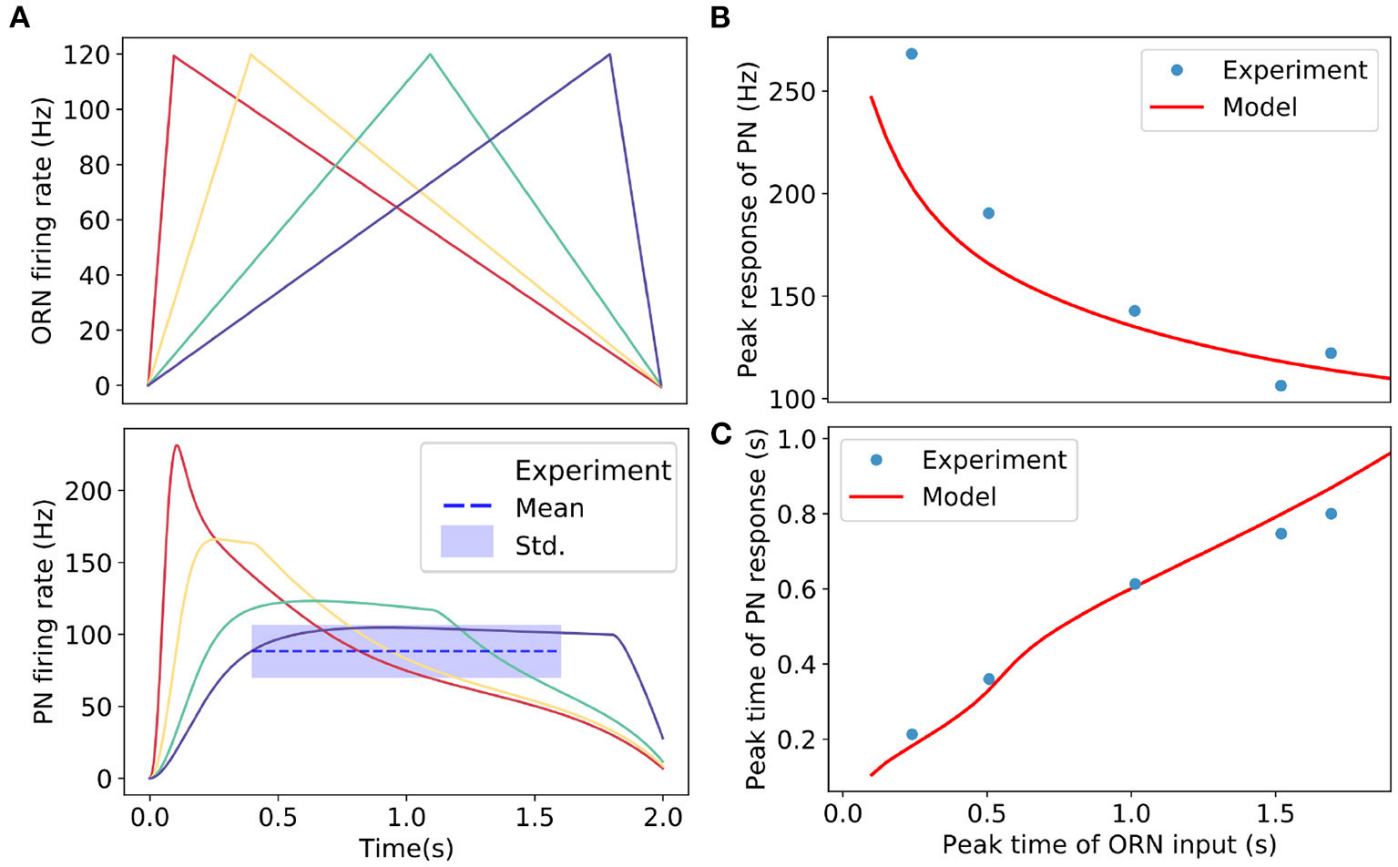
Adaptive responses of PN to triangle-shaped inputs (ORN spike rates). **(A)** Upper panel: Simulated triangle-shaped ORNs firing rates with different increasing rates in the rising phase. The peak inputs (ORN firing rates) are the same for all cases. Lower panel: responses of PNs to triangle-shaped ORNs inputs. For slow and medium increasing inputs, PNs reach plateau responses. The dotted line shows the average plateau response of PN estimated from experimental data with the shaded region indicating the standard derivation. **(B)** The peak responses of PNs increase with the rates of change in input signals. Our model result (solid line) agrees well with the experiment (dots). **(C)** PN's response reaches a peak earlier than that of the input signal. The model result (solid line) has an excellent agreement with the experiment (dots). Experimental results are from Kim et al. (2015). Parameters used in the model: *U* = 0.24, *R*_pub_ = 0Hz, k = 5Hz/nS, $\omega^{EE}=75nS,\omega^{IE}=21\text{nS},\tau_{E}=55\text{ms},\rho =8\text{ms},\tau_{p}=300\text{ms},\tau_{F}=50\text{ms},\tau_{D}=100\text{ms}$.

All model parameters were taken from previous studies or estimated by fitting to experimental data with their values constrained in reasonable physiological ranges. In Figure 2, each line is associated with a public odor at certain concentration. Since the firing rate due to all ORNs are not available in the experiment, and only local field potential was measured. We fitted each line by a independent ${\hat{R}}_{\text{pub}}$. The resulting ${\hat{R}}_{\text{pub}}$ are found proportional to local field potentials measured in experiments (Supplementary Figure 1). The best-fit parameters (Table 1) were obtained by minimizing the residual sum of square for all the 16 (DL5) or 15 (VM7) points for each ORN-PN pair. The qualitative behavior of modeling results in Figure 3 are robust across a wide range of parameters. The parameters used are based on parameters in Figure 2 with slight adjustment within physiological range. Parameters in Figures 4, 5 are the same, which are chosen to fit the plateau height, the peak response and peak time simultaneously. In all the numerical simulations of Equations (1)–(5), all the variables except *x* and *p* start from 0. *x* and *p*, according to their biological meanings introduced, all start from 1.

**Table 1:** Model parameters used in Figure 2.

**Parameter**	**Meaning**	**DL5**	**VM7**
τ_*E*_	Time constant for excitatory synapse	50 ms	50 ms
ω^*EE*^	Synaptic weight from ORN to PN	160 nS	105 nS
ω^*IE*^	Synaptic weight from ORN to LN	10 nS	10 nS
ρ	Intrinsic strength for presynaptic inhibition	1.9 ms	2.5 ms
U	Increase in release probability for faciliation	0.31	0.24
τ_*D*_	Time constant for STD	368 ms	160 ms
τ_*F*_	Time constant for STF	339 ms	150 ms

## 3. Results

### 3.1. STP Is Crucial for the Observed Nonlinear Divisive Normalization

A PN's response is suppressed by the firing of LNs, which can be activated by many ORNs, some of which can respond to a public odorant other than the cognate (private) odorant (see Figure 1). This introduces lateral inhibition and reduces the response of PN to a private odorant in the presence of public odorants. In Olsen et al. (2010), PN responses to a private odorant (which activates only the cognate ORN) with a background of different concentrations of a public odorant (presumably activates many other ORNs) were measured. As shown in Figure 2 (symbols are from experiments), the PN response to the private (cognate) odorant is affected by the level of public odorant (each color represents a different concentration of the public odorant). This divisive normalization effect was fitted phenomenologically by a Hill function in Olsen et al. (2010):

(11) $$\begin{array}{r} R_{PN}^{a}\approx R_{\text{max}}\frac{R^{\gamma_{a}}}{R^{\gamma_{a}}+K_{1/2}^{\gamma_{a}}+\sigma^{\gamma_{a}}}, \end{array}$$

where $R_{PN}^{a}$ is defined as the average response over the first 500ms after the stimulus is applied. Note that $R_{PN}^{a}$ is different from the adapted (steady state) response due to the finite adaptation time of PNs. In Equation (11), σ is proportional to the sum of the firing rates of non-cognate (public) ORNs; *R*_max_ is the maximum PN firing rate; *K*_1/2_ is the firing rate of ORN at which PN has the half maximum response when σ = 0, γ_*a*_ is the Hill coefficient for the average response. Olsen et al. (2010) were able to fit their experimental data with γ_*a*_ ≈ 1.5, which indicates a strong nonlinear effect in divisive normalization (γ_*a*_ > 1). However, the underlying mechanism of this “nonlinear” divisive normalization remains unclear.

Here, we use the circuit model to explain the experimentally observed nonlinear divisive normalization behaviors. By numerically solving our model (Equations 1–5), we computed the 500 ms average response $R_{PN}^{a}$ the same way as in the experiments for different public odorant backgrounds. As shown in Figure 2, our model fits well with the measured responses of both PNs that innervate either DL5 or VM7 glomeruli in different backgrounds of public odorant concentrations that are fitted as ${\hat{R}}_{\text{pub}}$ in the model. The fitted values of ${\hat{R}}_{\text{pub}}$ are found to be linearly proportional to the measured local field potentials in experiments (Olsen et al., 2010; see Supplementary Figure 1), which further supports the validity of our model. Other best-fit parameters are listed in Table 1. Most of the parameters for DL5 and VM7 remain approximately the same, however, the two STP timescales (τ_*D*_ and τ_*F*_) and the lateral inhibition strength ρ, which are intrinsic properties of the specific glomerulus, are different. Quantitatively, both STP timescales (τ_*D*_ and τ_*F*_) obtained from our model fitting are consistent with the range of these timescales measured in experiments (Abbott et al., 1997). We also find that the sensitivity to lateral inhibition (ρ) is slightly stronger of VM7 than that of DL5, which is also consistent with the experiments (Olsen et al., 2010).

The most interesting finding from fitting our model with the experimental data is that both DL5 and VM7 have strong STP effects (*S*_*F*_, *S*_*D*_ > 1), and both STF and STD strength are stronger in DL5 than those in VM7. However, the relative strength between STD and STF, *r* ≡ *S*_*D*_/*S*_*F*_, remains roughly the same for DL5 (*r* ≈ 1.08) and VM7 (*r* ≈ 1.07). Thus, our results suggest that strong and balanced STD and STF effects are responsible for the observed nonlinear divisive normalization in both VM7 and DL5. In fact, our model fails to fit experimental data without STP. As shown in Supplementary Figure 3, when we set τ_*D*_ = τ_*F*_ = 0, the PN response curves are roughly linear to ORN input within the range of the experimental data, which confirms the crucial role of STP in the observed “nonlinear” divisive normalization behavior.

To better understand this “nonlinear” effect in divisive normalization, we leveraged the simplicity of our model to derive an analytical expression for the steady state response of PNs. For any constant input (cognate ORN with firing rate *R*) to the antennal lobe, the output $R_{PN}^{*}\left(R\right)$, i.e., the firing rate of the cognate PN, can be determined analytically by solving the steady state in Equations (1–5):

(12) $$\begin{array}{r} R_{PN}^{*}\left(R\right)=\frac{\tau_{E}\omega^{EE}UR\left(\theta +\tau_{F}R\right)}{\theta^{2}+\theta \left(\tau_{F}+\tau_{D}\right)UR+\tau_{D}\tau_{F}UR^{2}}, \end{array}$$

where $\theta \equiv 1+A\sum_{j}R_{j}$ with $A\equiv \rho \omega^{IE}\tau_{E}$, and $\sum_{j}R_{j}=R+{\hat{R}}_{\text{pub}}$ with ${\hat{R}}_{\text{pub}}$ denoting the total input from public ORNs.

In the absence of STP, i.e., when τ_*D*_ → 0 and τ_*F*_ → 0, *x* = 1 and *u*^+^ = *U* remain constant, the PN response (Equation 12) reduces to:

(13) $$\begin{array}{r} R_{PN}^{*}\left(R\right)=\tau_{E}\omega^{EE}U\frac{R}{1+A\sum R_{j}}, \end{array}$$

where both the numerator and the denominator depend linearly on *R*. We thus refer to Equation (13) as “linear” divisive normalization. In the presence of STP, i.e., when τ_*F*_ ≠ 0 and τ_*D*_ ≠ 0, both the denominator and the numerator in Equation (12) are nonlinear in *R*, which leads to the “nonlinear” divisive normalization behavior.

Similar to the empirical Hill function (11), the above steady state PN response curve exhibits a sigmoidal shape that can be characterized by three parameters: the maximum response $R_{\text{PN}}^{\text{max}}\equiv R_{\text{PN}}^{*}\left(R=\infty \right)=\frac{\tau_{E}\omega^{EE}U\left(A+\tau_{F}\right)}{A^{2}+A\left(\tau_{F}+\tau_{D}\right)U+U\tau_{F}\tau_{D}}$; the half maximum input *R*_1/2_ defined as $R_{PN}^{*}\left(R_{1/2}\right)=R_{\text{PN}}^{\text{max}}/2$; and an effective Hill coefficient $\gamma \equiv 2\frac{d\ln\left(R_{\text{PN}}^{*}\right)}{d\ln\left(R\right)}{|}_{R_{1/2}}$. From (13), we have the linear divisive normalization behavior (γ = 1) in the absence of STP. In the presence of STP, we have γ > 1, which can be used to characterize the nonlinearity of the response. In Figures 3A,B, the PN response function (12) with different STP strengths are shown. As expected, for a given input *R*, STF enhances the response while STD suppresses it, and the latter has a stronger effect. The dependence of γ on the STP strengths *S*_*D*_ and *S*_*F*_ are shown in Figure 3C. Interestingly, the Hill coefficient γ is enhanced by both STD and STF. Larger values of γ are reached by having roughly the same STD and STF strengths (dotted line in Figure 3C). A careful comparison between the Hill coefficient γ_*a*_ obtained using 500 ms average response of PNs (as in the experiments) and the effective Hill coefficient γ from the steady state response showed that they are highly correlated and both show similar dependence on *S*_*F*_ and *S*_*D*_ (Supplementary Figure 2). Thus, the steady state analysis confirms the crucial role of STP in generating the experimentally observed nonlinear divisive normalization.

### 3.2. Both STP and PI Control the Adaptive Responses to Time-Varying Stimuli

Odors in the environment are highly intermittent and dynamic (Murlis et al., 1992; Vickers et al., 2001). The ability to detect and respond to temporal variation of odor stimuli is crucial for the survival of many animals. Kim et al. (2015) studied the responses of PNs to different time-dependent ORN signals that follow triangle-shaped temporal patterns with different peak times. The responses of PNs were “asymmetric,” with a faster rising phase, followed by a plateau and a slower decaying phase, depending on the rate of change in ORN's firing rate profile (see Figure 3 in Kim et al., 2015). Here, we use our model to explain the response patterns to these time-dependent signals.

We start by considering the response of PNs to a signal that increases linearly with time, i.e., *R*(*t*) = *Kt*. In the short time limit *t* ≪ τ_*p*_, PI is negligible so the reduction factor *p* ≈ 1 (see Equation 3). As a result, the response is linearly proportional to the input $R_{\text{PN}}\left(t\right)\approx \tau_{E}\omega^{EE}UKt$. In the long time limit *t* ≫ τ_*p*_, τ_*E*_, *p* ≈ (*AR*(*t*))^−1^ decreases inversely proportional to the input *R*(*t*) which reduces the effective input *pR*(*t*). In fact, the effective input can be approximated as (see section 2 for detailed derivation):

(14) $$\begin{array}{r} R_{\text{eff}}\equiv pR\left(t\right)\approx \frac{Kt}{AK\left(t-\tau_{E}\right)+1}-\frac{AK^{2}\tau_{E}t}{1-AK\tau_{E}}\cdot e^{-\frac{t}{\tau_{p}}}. \end{array}$$

In the long time limit *t* ≫ τ_*E*_, τ_*p*_, the system adapts by adjusting the inhibition factor *p* so that the effective input reaches a constant $R_{\text{eff}}^{*}=A^{-1}={\left(\rho \omega^{IE}\tau_{E}\right)}^{-1}$ as *t* → ∞, which is independent of the input. The corresponding adapted response can thus be determined analytically:

(15) $$\begin{array}{r} R_{\text{PN}}^{*}\approx \frac{\tau_{E}\omega^{EE}UR_{\text{eff}}^{*}\left(1+\tau_{F}R_{\text{eff}}^{*}\right)}{1+UR_{\text{eff}}^{*}\left(\tau_{F}+\tau_{D}\right)+U\tau_{D}\tau_{F}{R_{\text{eff}}^{*}}^{2}}, \end{array}$$

which takes exactly the same form as the response to an effective time-independent signal $R_{\text{eff}}^{*}$ as in (12). This is supported by numerical simulation as shown in Figure 4. The effect of PI is crucial for canceling out the increasing signal, resulting in a constant effective input $R_{\text{eff}}^{*}$. From Equation (15), it is clear that STP controls (modulates) the adapted response $R_{\text{PN}}^{*}$ in the same way as it affects the response to a constant signal, i.e., STF enhances the adapted response and STD suppresses it.

We now study the PN responses to triangle-shaped input signals similar to those used in experiments (Kim et al., 2015) with our model. As shown in Figure 5A, the general response dynamics follows closely the experimentally observed behaviors. During the rising phase of the input signal, the PN response activity reaches its peak in a timescale that depends on the rate of change (*K*) of the input signal. For small *K* shown as the purple line in Figure 5A, the PN response reaches a plateau before the input reaches its peak due to the adaptive effect of presynaptic inhibition described above. The shaded region in Figure 5A (lower panel) shows the range of the experimentally observed plateau consistent with the model result (purple line). Note that for higher *K* the plateau activities depend on the rates of change in the input signals because they do not have enough time to reach the adapted value. In the descending phase of the input signal, the PN activity decreases following the input.

Quantitatively, the PN response can be described by two parameters: the peak response, which is defined as the PN activity at the peak time of the input signal, and the time to reach the peak response. In Figures 5B,C, we show that the results for these quantities measured from our model are in excellent agreement with those obtained from experiments (Kim et al., 2015).

Interestingly, STP alone can also lead to adaptive responses. As shown in Figure 1B, aside from the IFFL in which the negative (inhibitory) arm is formed via presynaptic inhibition (PI) by LN (*ORN* → *LN* → *PN*), there is another IFFL in the circuit with the negative (inhibitory) feedforward arm formed via STD (*ORN* → *x* → *PN*), which can achieve adaptive response even in the absence of PI. Quantitatively, for the experimental system studied by Kim et al. (2015), we find that the adaptive response is more strongly affected by PI. This can be seen by comparing PN's response to triangle-shaped input in three model variants: the standard model used in Figure 5 with strong PI and moderate STP, a model with PI only and without STP (*S*_*D*_ = *S*_*F*_ = 0), and a model with only STP without PI (ρ = 0). As shown in Supplementary Figure 4, the model response agrees with experiments qualitatively well even with PI alone, however, STP is required to achieve quantitative agreement with experiments. On the other hand, even though a very strong STD may also cause the system to exhibit adaptive response (Supplementary Figure 4B), the fit of the STP-only model to experimental data remains relatively poor even with fine tuning of the STF and STD strengths (see Supplementary Figures 4B–D for details).

Our results show that both STD and PI control the adaptive response albeit via different routes for the feed-forward inhibition and both of them are needed to explain the experimental results quantitatively. Given their different timescales and the fact that the PI mediated inhibition occurs upstream of the inhibition caused by STD, the more dominant role of PI suggested by our analysis seems reasonable. However, we believe that the double IFFL design in the AL circuit may represent a network architecture that improves the robustness of the system and STP is needed to modulate the adapted activity [see Equation (15)].

## 4. Discussion

In this study, we developed a simple neural circuit model for the antennal lobe of *Drosophila* and systematically studied the role of STP in early odor information processing. Combining analytical derivations of a steady-state solution of the model and numerical simulations, we showed that the model can capture key characteristics of PNs' responses to different ORN inputs, in particular divisive normalization and adaptive responses to time-varying signals. Comparison with experimental results revealed that STP is crucial for the observed nonlinear divisive normalization. We found that there are two IFFLs (one via STP and the other via PI) in the AL circuit (Figure 1B) and either of the two IFFLs or together can lead to adaptive response. We speculate that this sequential IFFL design in the AL circuit may be desirable for a robust adaptive behavior. Since both divisive normalization and adaptive response to time-varying inputs are omnipresent in nervous systems, the effects of STP identified here may also apply to other sensory modalities and brain regions.

Previous experiments have suggested that STD largely determines the nonlinear response function of PNs (Kazama and Wilson, 2008), and PI plays an important role in divisive normalization (Olsen and Wilson, 2008; Olsen et al., 2010). Our model extends these studies and showed that STP is crucial for the observed nonlinear divisive normalization. For stable ORN input, STF enhances PN's response while STD suppresses it. Yet, both STF and STD enhance the nonlinearity of the divisive normalization (Figure 3). Although the transient response of PNs to step-like ORN input has been attributed to STD, our model shows that PI predominately determines PN's response to more dynamic ORN input, such as triangle-shaped input patterns (Figure 5). In fact, our model can predict the response properties of PNs to arbitrary time-dependent ORN inputs. For example, when applied to a set of sine-wave ORN inputs with different frequencies, our model predicts that the amplitude of PN's oscillatory response will increase as the frequency gets higher, while the time advance of PN response peak to ORN response peak will decrease (see Supplementary Figure 5). These predictions can be tested by future experiments.

Our model not only reveals the underlying mechanisms for the observed nonlinear divisive normalization behavior and adaptive responses to time-varying signals, it also provides a general framework for relating the microscopic properties of the system such as time scales and strengths of STP and PI to macroscopic behaviors such as the input-output relation. As demonstrated in this work in the cases of VM7 and DL5 glomeruli, we can use our model to infer microscopic properties of the system from the measured input-output relation. More specifically, from our model study, the timescales of both STD (τ_*D*_) and STF (τ_*F*_) are predicted to be longer in DL5 than those in VM7, which can be verified by experiments (Abbott et al., 1997). The model-based analysis of the input-output response can be extended to other glomeruli. Our model can also be used to make predictions for changes in the input-output relation when certain microscopic properties, e.g., the STP strength (τ_*F*_ and τ_*D*_) or the PI strength (ρ) are perturbed. These predictions can be tested in future experiments.

As we focused on building a minimal model to understand the underlying mechanism for nonlinear divisive normalization and adaptive response, we have made several simplifications in our study. First, our model is a mean-field rate model which neglects the noise. The nonlinear divisive normalization effect is defined based on steady state firing rate of PN (Equation 12), which only depends on the averaged values of variables in Equations (1)–(5). Additive noise (with 0 mean) in the neural dynamics of PN and LN will be averaged out and does not affect the steady state firing of PNs. For the adaptive response of PNs to time-varying stimuli, additive noise does not change the qualitative behavior of peak time, peak firing rate and plateau firing rate of PNs (Supplementary Figure 7).

Second, at the synapse level, our model ignores the STP effect at the ORN-LN synapses (Nagel and Wilson, 2016), which can affect the LN response especially its temporal dynamics. In our circuit model, we only considered one type of LNs for simplicity. There are several types of LNs in the antennal lobe with diverse innervation patterns and physiological properties (Chou et al., 2010; Nagel et al., 2015; Nagel and Wilson, 2016). For example, a small fraction of LNs are excitatory. Panglomerular LNs innervate all glomeruli and have higher spontaneous firing rates than other LNs. They are inhibited or only weakly excited by odors. Such inhibition of panglomerular LNs tends to dis-inhibit the entire AL in the presence of odors (Chou et al., 2010). LNs also show distinct response dynamics to odors. This temporal diversity of LNs likely shape the dynamics of PNs to complex ecologically relevant odor stimuli, such as the response time and the synchronization of spikes across PNs (Tanaka et al., 2009; Nagel and Wilson, 2016). Flies can sense temporal features of odor plume to help navigation (Álvarez-Salvado et al., 2018; Demir et al., 2020). Future study should address how the additional complexity of AL circuit affect PN's response properties and contribute to the olfactory behavior (Kao and Lo, 2020). Furthermore, we only considered PNs that innervate a single glomerulus (uPNs), some PNs do receive input from multiple glomeruli (mPNs). uPNs and mPNs have different projection patterns and may carry different aspects of odor information to the higher brain regions such as lateral horn and mushroom body (Bates et al., 2020). Future studies that incorporate these important features (both at the synapse level and the network level) will further our understanding of the rich dynamics in the early olfactory information processing in *Drosophila*.

## Data Availability Statement

The original contributions presented in the study are included in the article/Supplementary Material, further inquiries can be directed to the corresponding author/s.

## Author Contributions

YL, QL, SQ, and YT contributed to the study design and prepared the manuscript. YL performed all simulations. CT and YT supervised the study. All authors read and approved the final manuscript.

## Funding

The work was supported by the National Natural Science Foundation of China (Grant Nos. 12090053 and 32088101). The work by YT was supported by a NIH grant (R35GM131734).

## Conflict of Interest

YT was employed by IBM T. J. Watson Research Center. The remaining authors declare that the research was conducted in the absence of any commercial or financial relationships that could be construed as a potential conflict of interest.

## Publisher's Note

All claims expressed in this article are solely those of the authors and do not necessarily represent those of their affiliated organizations, or those of the publisher, the editors and the reviewers. Any product that may be evaluated in this article, or claim that may be made by its manufacturer, is not guaranteed or endorsed by the publisher.
