# Supplementary material for: Short-Term Plasticity Regulates Both Divisive Normalization and Adaptive Responses in Drosophila Olfactory System
Source: Front Comput Neurosci. 2021 Oct 22;15:730431. doi: 10.3389/fncom.2021.730431 (PMC8568954; doi:10.3389/fncom.2021.730431)
Supplement: Supplementary file 1 [file Data_Sheet_1.PDF]

Supplementary Material

1 SUPPLEMENTARY FIGURES

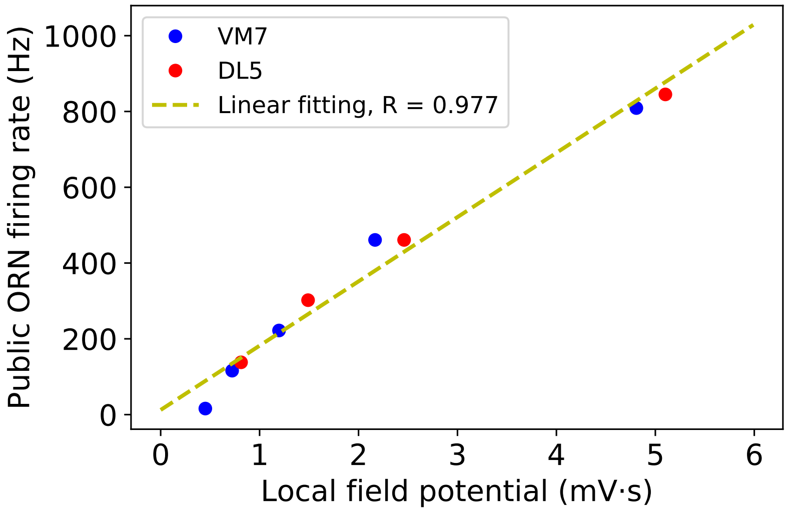

Figure S1. Fitted public ORN firing rates $\hat{R}_{\mathrm{pub}}$ (see Figure 2 in the main text) are proportional to the measured local field potentials in experiments Olsen et al. (2010).

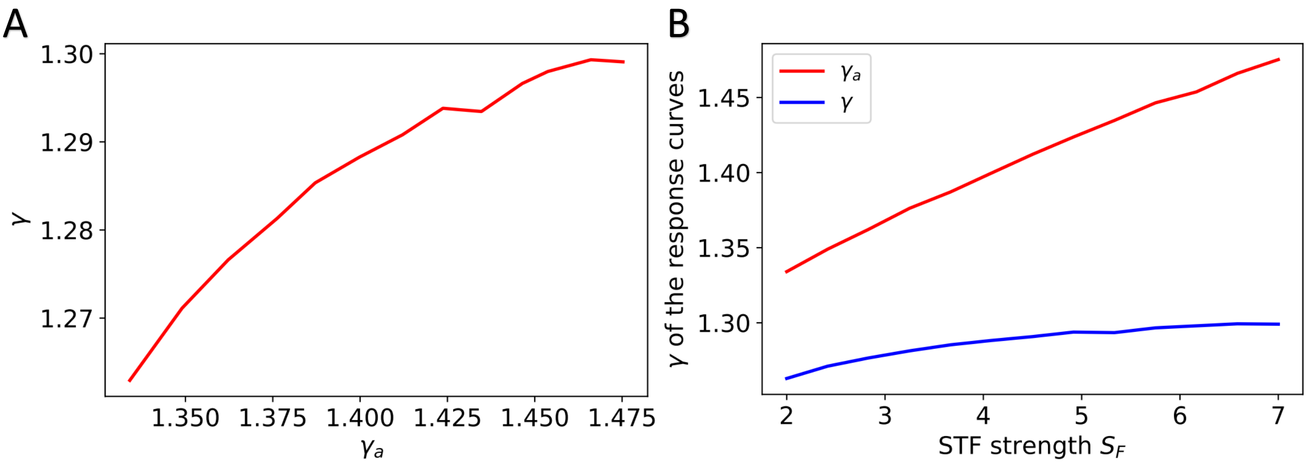

Figure S2. Comparison of Hill-coefficient derived from steady state PN responses (γ) and from average firing rate over 500 ms stimulus period (γ_a). **(A)** γ and γ_a are positively correlated. **(B)** The dependence of γ and γ_a on STP strength. We calculate the dependence along the line of $S_D = 1.07S_F$, which is determined by that the fitted τ_D and τ_F of both DL5 and VM7 data have approximately such relationship. Other parameters used for the simulation: $U = 0.27, \omega^{EE} = 160nS, \omega^{IE} = 10\mathrm{nS}, \tau_{\mathrm{E}} = 50\mathrm{ms}, \rho = 2\mathrm{ms}, \tau_{\mathrm{p}} = 30\mathrm{ms}$.

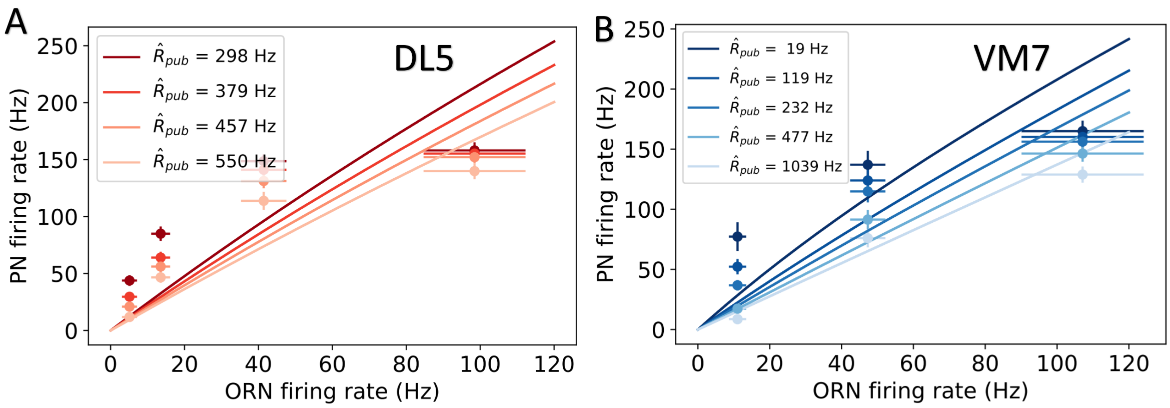

Figure S3. The best fit of the model (lines) to the experimental data (symbols) Olsen et al. (2010) without STP ($\tau_D = \tau_F = 0$). In the absence of STP, the predicted responses are almost linearly dependent on the input (ORN firing rate), which does not agree with the experimental data for either DL **(A)** or VM7 **(B)**.

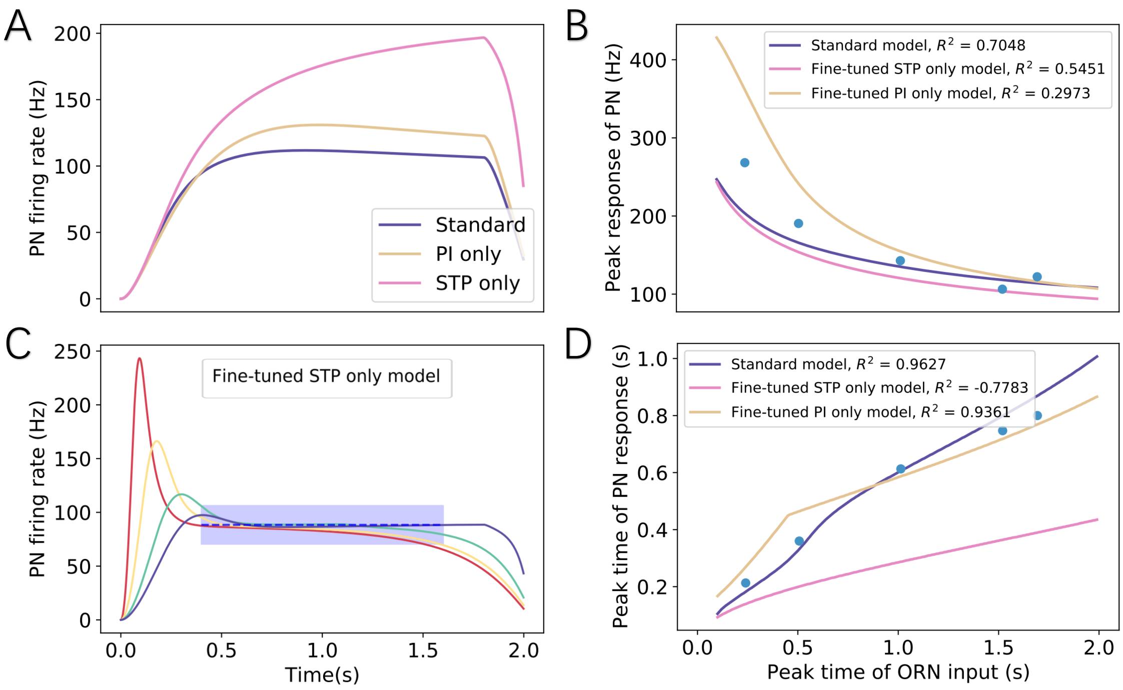

Figure S4. PN response under different conditions reveals the interaction between STP and presynaptic inhibition (PI). **(A)** PN response in three model variants to triangle-shaped ORN input as in Figure 5 with peak time at 1.8 s and peak amplitude 120 Hz. All the three models share the same set of parameters as in Figure 5, except that the PI only model has $\tau_D = \tau_F = 0$ and the STP only model has $\rho = 0$. **(B)** The best fit to the experiments by using the STP only model. The parameters are selected to best fit the plateau height (shaded region) for the slowest input. Notice that the STP-only model can not distinguish the experimentally observed different plateau heights for different inputs. Comparing the fitting of three model variants (standard, STP only, and PI only) with experimentally observed peak response **(C)** and peak response time **(D)**, we found the standard model performs much better than other model variants. Note that the R^2 value for the STP only model is negative, which means that the STP-only model fails to fit the peak time data when it is tuned to fit the plateau height as shown in **(D)**. Parameters in the variant models that are different from the standard model are: $\tau_D = 270$ ms, $\tau_F = 100$ ms, $\rho = 0$, $U = 0.2$, $\omega^{EE} = 90$ nS for the STP-only model; $\tau_D = 0$ ms, $\tau_F = 0$ ms, $\rho = 10$ ms, $\tau_p = 250$ ms, $U = 0.25$, $\omega^{EE} = 80$ nS for the PI-only model.

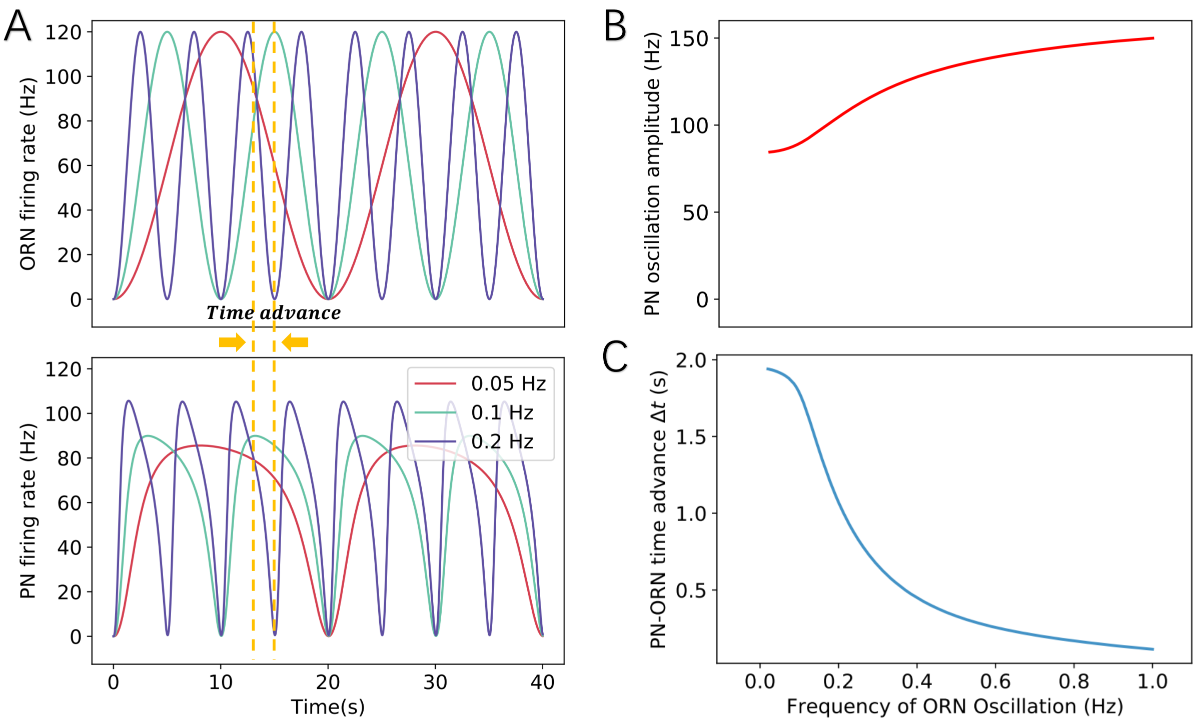

Figure S5. Prediction of PN response to oscillatory ORN inputs. **(A)** Upper panel: Simulated sine-wave ORNs firing rates with different frequency. The peak firing rates are the same for all inputs. Lower panel: responses of PNs to sine-wave ORNs inputs. The two dotted lines mark the peak of ORN input and its corresponding PN output with a frequency of 0.1 Hz. The gap between these dotted lines is defined as the time advance Δt of PN's response. **(B)** The peak responses of PNs increase with the frequency of ORN oscillation. **(C)** PN's response always reaches to a peak earlier than that of the input signal, but the time advance Δt decreases as the oscillation frequency increase. Parameters used in the model are the same as in Figure 5 in the main text.

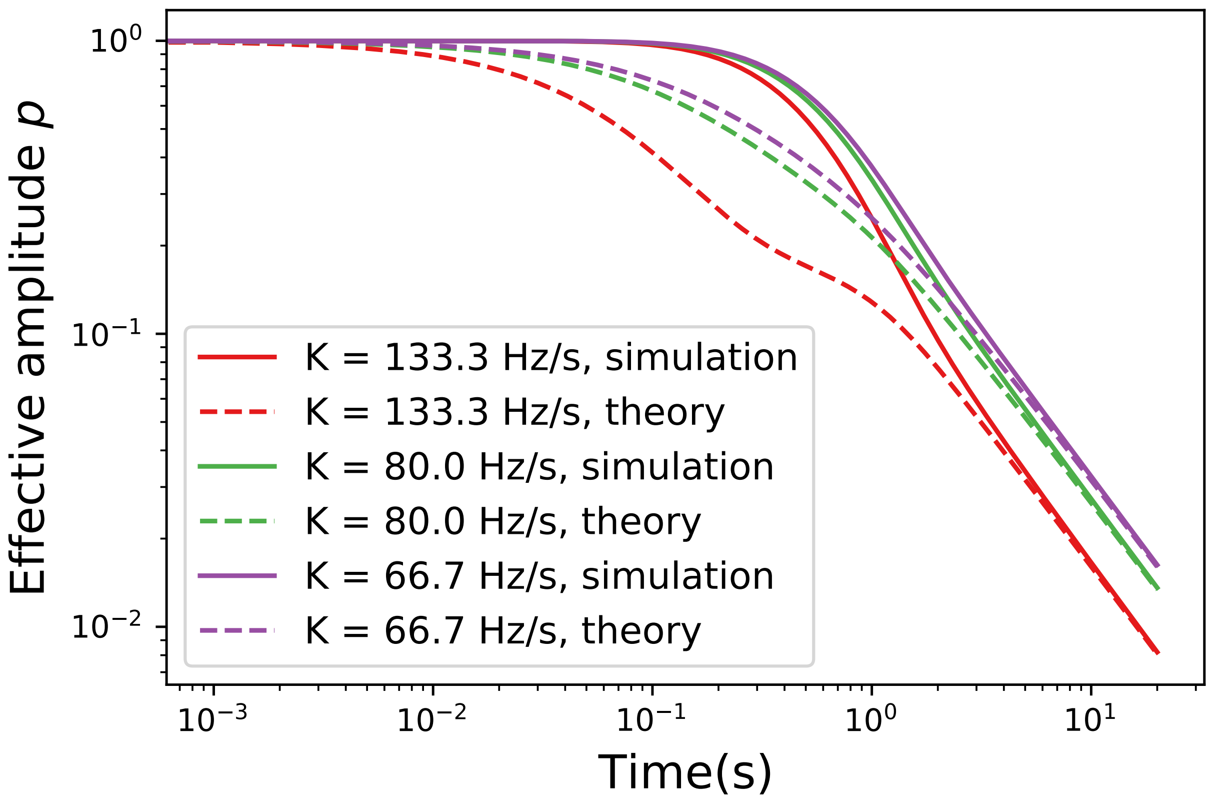

Figure S6. Comparison of direct numerical simulation results of the effective amplitude p and approximate analytical results (Eq.(7) in the main text) for 3 sets of increasing rates (K) under log-log axises. Other parameters used are exactly the same as those in Fig.4 in the main text.

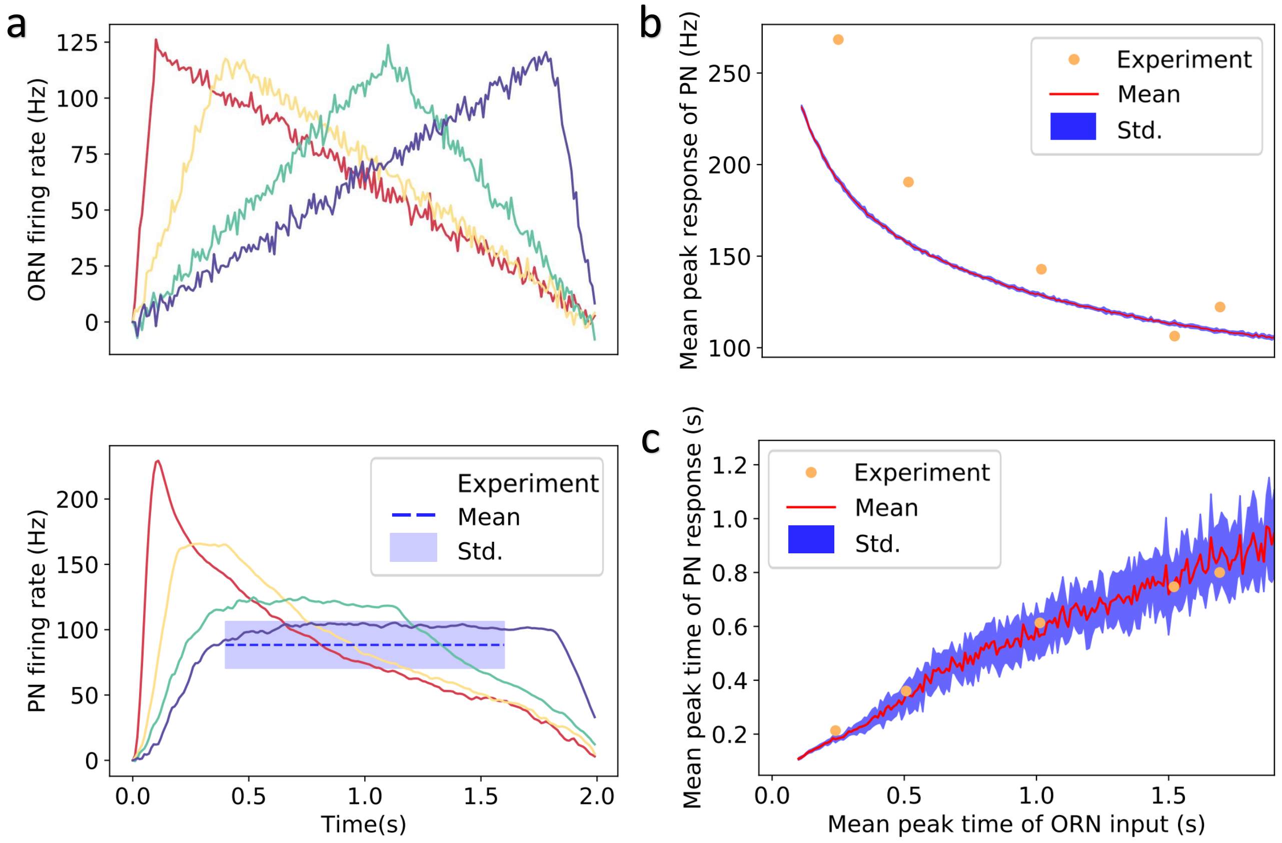

Figure S7. PN responses in the presence of noise (noisy ORN inputs). (a) Examples of simulation results. Upper panel: noisy ORN inputs. Lower panel: PN responses. Gaussian white noise with zero mean and a standard deviation of 3Hz is added to the ORN input. (b) Average of 20 simulations of PN response peak values. (c) Average of 20 simulations of PN response peak times. All parameters used for simulation except the additional noise are the same with those in Fig.5 in the main text.

REFERENCES

Olsen, S. R., Bhandawat, V., and Wilson, R. I. (2010). Divisive normalization in olfactory population codes. *Neuron* 66, 287–299
